# Spontaneous gene mutation encounters a paternal genetic variant: Nemaline myopathy: a case report and literature review

**DOI:** 10.1097/MD.0000000000051064

**Published:** 2026-10-16

**Authors:** Yan-Fang Zhang, Guo-De Wu, Ya-Zhen Hao, Wen-Juan Li, Xiao-Ling Qian, Jian-Fei Wang

**Affiliations:** a Department of Neurology, The Second Hospital of Lanzhou University, Lanzhou, China; b The Second Clinical Medical College, Lanzhou University, Lanzhou, China; c Department of Cardiology, The Second Hospital of Lanzhou University, Lanzhou, China.

**Keywords:** congenital myopathy, genetic testing, heterozygous mutation, nemaline myopathy

## Abstract

**Rationale:**

Nemaline myopathy (NM) is a rare hereditary myopathy, most commonly caused by mutations in the *NEB* gene. This case highlights the diagnostic challenges and expands the mutational spectrum of the disease.

**Patient concerns:**

A 21-year-old male presented with a history of progressive muscle weakness. The symptoms began in the distal lower limbs at age 3 and later involved proximal and masticatory muscles.

**Diagnoses:**

The patient was diagnosed with nemaline myopathy type 2. Diagnosis was based on electromyography suggesting myogenic damage, muscle magnetic resonance imaging (MRI) showing extensive atrophy and fatty infiltration, muscle biopsy revealing nemaline rods, and genetic testing confirming compound heterozygous *NEB* mutations (paternal c.21417 + 3A > G and de novo c.7272G > A).

**Interventions:**

The patient received symptomatic treatment.

**Outcomes:**

Symptomatic treatment provided limited clinical improvement. This is the first report confirming the pathogenicity of the *NEB* c.7272G > A mutation.

**Lessons:**

Genetic testing is essential for diagnosing NM. Clinicians should be aware of NM subtypes that initially present with masticatory muscle weakness. This case provides new evidence for genotype-phenotype correlation and is valuable for genetic counseling.

## 1. Introduction

Nemaline myopathy (NM) is a rare hereditary myopathy named after the presence of nemaline rods within muscle fibers. Clinically, it is primarily characterized by proximal limb and trunk muscle weakness and atrophy, which may progress to generalized weakness, often accompanied by facial, lingual, and pharyngeal muscle involvement. According to the age at onset and degree of muscle weakness, the following 6 types of NM have been clinically defined by the European Neuromuscular Center International Consortium on NM: the severe congenital form, the intermediate congenital form, the typical congenital form, childhood-onset NM, adult-onset NM, and other forms of NM with unusual associated features, such as cardiomyopathy or ophthalmoplegia.^[1,2]^

However, this classification method has a weak correlation between genotype and phenotype. Therefore, Laitila et al^[3]^ proposed a new classification method, in which the severe type has intrauterine onset, presenting with joint contractures, respiratory distress, fractures, etc; the classic type has perinatal onset, presenting with motor developmental delay; the mild type has onset in childhood or adolescence; the distal type only presents with distal muscle weakness; other uncommon types present with abnormal distribution of muscle weakness. The *NEB* gene can cause all of the above types.^[3]^

At present, a total of 13 causative genes have been implicated, namely *NEB, ACTA1, TPM2, TPM3, TNNT1, KBTBD13, CFL2, KLHL40, KLHL41, MYO18B, LMOD3, TNNT3*, and *MYPN*.^[4]^ Among these, *NEB* and *ACTA1* are the 2 most commonly mutated loci. *NEB* mutations are typically compound heterozygous mutations with autosomal recessive inheritance; however, it has recently been discovered that a family’s *NEB* exhibits a dominant mutation inheritance pattern.^[5]^ Nemaline myopathy type 2 (NM2), caused by *NEB* mutations, represents the most common subtype and demonstrates a broad phenotypic spectrum, ranging from neonatal respiratory failure to slowly progressive muscle weakness in adolescence or adulthood.^[6]^ However, the relationship between specific *NEB* variants and clinical severity remains incompletely understood. Diagnosis primarily relies on muscle biopsy and genetic testing.

Herein, we reported a patient with NM2 caused by a compound heterozygous *NEB* mutation, including a **de novo** mutation not previously reported, and reviewed the previous studies. This case expands our understanding of genotype–phenotype correlation and provides new insights into the mutational spectrum of *NEB*.

## 2. Case presentation

### 2.1. Initial presentation and clinical history

A 21-year-old Hui male was admitted to The Second Hospital of Lanzhou University on January 10, 2024, with an 18-year history of progressive lower limb weakness and a 12-year history of masticatory weakness, both of which had worsened over the preceding year. He had an uneventful perinatal period and normal early infant development. At age 3, he developed bilateral lower limb weakness without an obvious trigger, manifested as unsteady gait, heel-strike walking, in-toeing, and frequent falls, and his lower limbs appeared thinner than those of his peers. At age 9, he developed left foot inversion, adduction, and equines deformity, with difficulty squatting, rising, and chewing hard food. He could not run, jump, or climb stairs independently, and no medical care was sought at that time. At age 18, he was diagnosed with congenital talipes equinovarus at our hospital and underwent tibiotalar arthrodesis, after which his condition remained stable with independent ambulation. During the past year, his lower limb weakness worsened and impaired his daily activities. Apart from the ankle correction surgery described above, his past medical history was unremarkable.

### 2.2. Personal and family history

The patient denies any history of smoking or alcohol use. A detailed 3-generation pedigree was constructed (Fig. 2). The patient was the only affected individual in the family. Neither parent nor other available relatives exhibited clinical manifestations suggestive of NM, including muscle weakness, respiratory insufficiency, facial involvement, or swallowing dysfunction. His parents are non-consanguineous. There was no history of recurrent miscarriage, stillbirth, or unexplained neonatal death.

**Figure 1.**
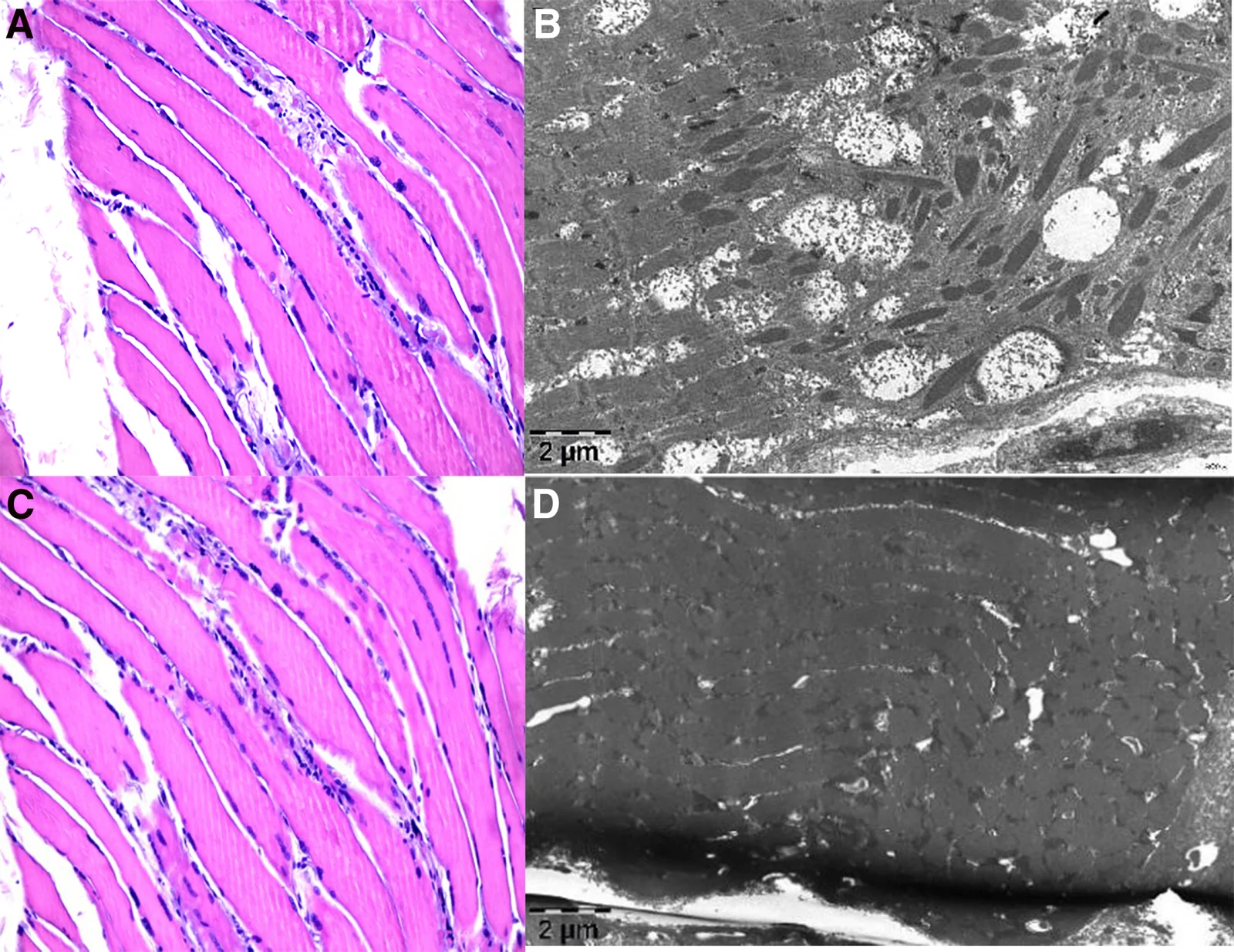
**Light and electron microscopy features of nemaline myopathy (NM).** (**A**) Gastrocnemius hematoxylin and eosin (HE) staining showed variable fiber size with fatty infiltration, partial fiber necrosis, and abnormal red-staining deposits (HE × 200). (**B**) Electron microscopy of gastrocnemius showed focal rod aggregation in muscle cells, rods > 2000 nm, and occasional subsarcolemmal lipofuscin granules (×9900). (**C**) Triceps brachii HE staining showed variable fiber size with fatty infiltration, without abnormal red deposits (HE × 200). (**D**) Electron microscopy of triceps brachii revealed no rods, with occasional subsarcolemmal lipofuscin granules.

**Figure 2.**
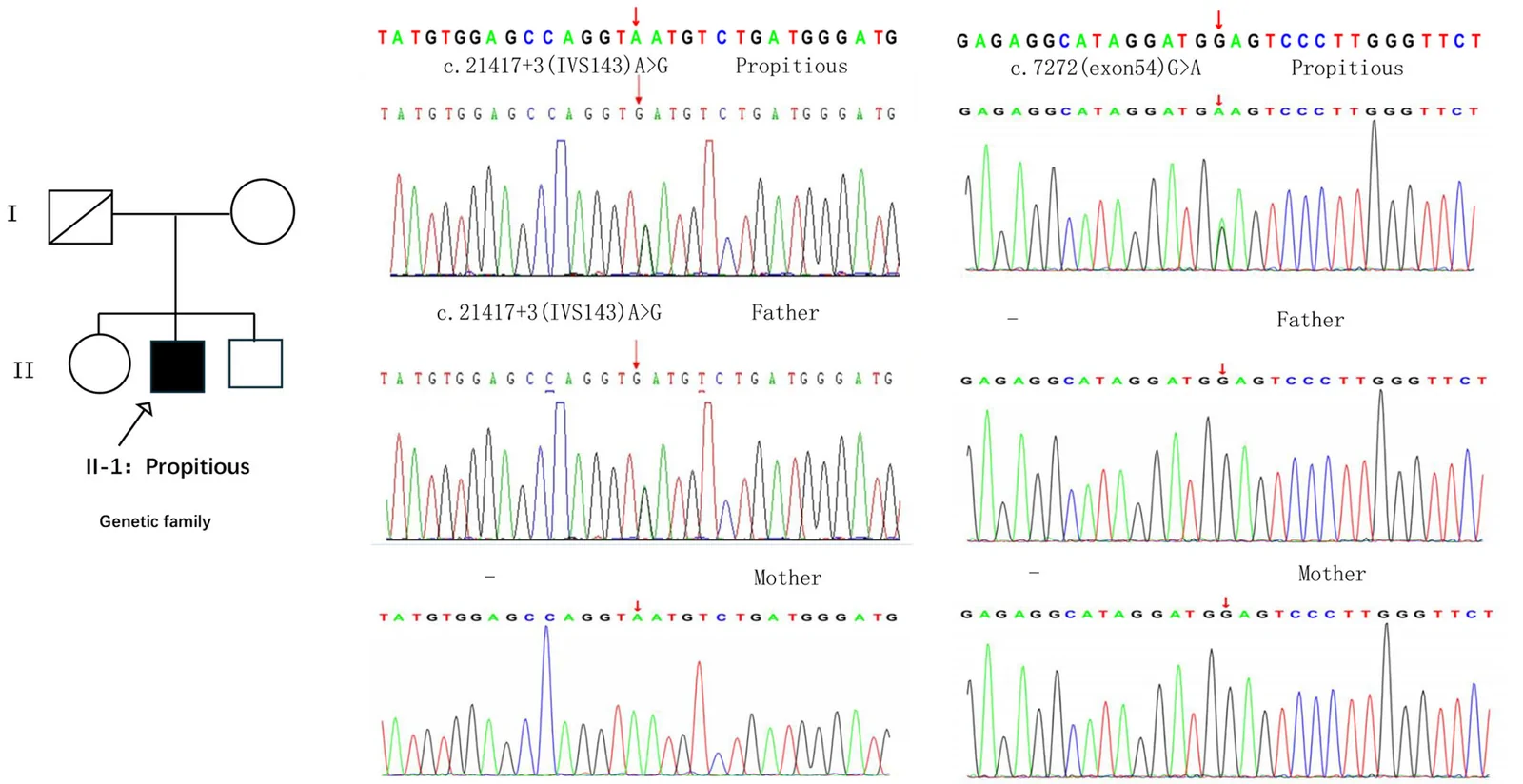
Pedigree and genetic analysis. The proband carried compound heterozygous mutations in the *NEB* gene: c.21417 + 3 (IVS 143) A > G and c.7272 (exon 54) G > A.

### 2.3. Neurological examination

The patient was alert with fluent speech and a long narrow face. Bilateral masticatory weakness and delayed gag reflex were observed. Neck flexion strength was grade 5, upper limb strength grade 5, proximal lower limb strength grade 4^−^, and distal lower limb strength grade 3 with foot drop. Marked lower limb muscle atrophy, normal tone, absent knee and ankle reflexes, and no pathological signs were noted. The feet showed equinovarus deformity with high arches, and he exhibited a steppage gait.

### 2.4. Laboratory examinations

Thyroid function, liver function, hepatitis virus serology, syphilis, human immunodeficiency virus, folate, vitamin B12, homocysteine, immunological markers, and tumor markers were normal. Creatine kinase was 63 U/L (reference: 50–310 U/L), and creatine kinase-MB (CK-MB) was 12 U/L (reference: <24 U/L).

### 2.5. Imaging examinations

Muscle magnetic resonance imaging (MRI) showed extensive fatty atrophy of thigh and calf muscles, most prominent in soleus and medial gastrocnemius. Correspondingly, electromyography yielded findings consistent with myogenic involvement.

### 2.6. Histopathology

A muscle biopsy of the gastrocnemius, stained with hematoxylin and eosin (HE), demonstrated fiber size variation, fatty infiltration, focal fiber necrosis, and abnormal red deposits (Fig. 1A). Ultrastructural examination by electron microscopy revealed focal aggregates of nemaline rods exceeding 2000 nm in length, along with occasional subsarcolemmal lipofuscin deposits (Fig. 1B). In contrast, a biopsy of the triceps showed fiber size variation and fatty infiltration but no abnormal deposits (Fig. 1C), and electron microscopy confirmed the absence of rod structures (Fig. 1D).

### 2.7. Gene mutation testing

Three milliliters of peripheral venous blood were collected from the patient and both parents into tubes containing ethylenediaminetetraacetic acid (EDTA) as anticoagulant. Genomic DNA was extracted from peripheral blood leukocytes using the QIAamp DNA Blood Mini Kit (Qiagen, Hilden, Germany) according to the manufacturer’s instructions. The extracted DNA samples underwent quality control using a Qubit 2.0 fluorometer (Thermo Fisher Scientific, Waltham) and electrophoresis on a 1% agarose gel before further processing.

Protein-coding exome enrichment was performed using xGen Exome Research Panel v2.0 (IDT, Coralville) that consists of 429,826 individually synthesized and quality-controlled probes, which targets 39 Mb protein-coding region (19,396 genes) of the human genome and covers 51 Mb of end-to-end tiled probe space.

High-throughput sequencing was performed on an MGISEQ-T7 series sequencer (BGI, Shenzhen, China), and not <99% of the target sequence was sequenced. Sequencing was carried out by Beijing Chigene Translational Medicine Research Center Co., Ltd. (Beijing 100875, China). Primers were designed for candidate pathogenic variants based on the sequencing results, and Sanger sequencing was performed for familial verification.

### 2.8. Bioinformatics analysis

Raw data were processed using fastp for adapter removal and low-quality read filtering. Alignment of the paired-end reads to the GRCh37/hg19 human reference genome was performed using Burrows-Wheeler Aligner. Base quality score recalibration together with single nucleotide polymorphism (SNP) and short insertion/deletion calling was conducted using the Genome Analysis Toolkit. According to the sequence depth and variant quality, SNPs and insertion/deletions were screened to obtain high-quality and reliable variants.

### 2.9. Variant annotation and pathogenicity prediction

The online system independently developed by Chigene (www.chigene.org) was used to annotate database-based minor allele frequencies (MAFs) and to interpret the pathogenicity of every yielded gene variant according to the American College of Medical Genetics and Genomics (ACMG) practice guidelines.^[7]^ This system also provides a series of software packages for conservation analysis and protein product structure prediction. The databases used for MAF annotation include 1000 Genomes, dbSNP, ESP, ExAC, and the Chigene in-house MAF database. PROVEAN, SIFT, PolyPhen-2 (HDIV and HVAR), MutationTaster, M-CAP, and REVEL were used to predict protein product structure variation. As a prioritized pathogenicity annotation to the ACMG guidelines, the OMIM, HGMD, and ClinVar databases were used as references for the pathogenicity of every variant. To predict the functional consequences of variants at splice sites, MaxEntScan, dbscSNV, and GTAG were used instead of protein structure prediction software.

### 2.10. Genetic test results

Peripheral venous blood samples were collected from the patient and both parents for whole-exome sequencing after written informed consent had been obtained from the patient and both parents. Genetic analysis identified 2 heterozygous variants in the *NEB* gene: c.21417 + 3A > G at chromosome 2:152389953 and c.7272G > A at chromosome 2:152506849. Sanger sequencing confirmed that the c.21417 + 3A > G variant was inherited from the patient’s father, whereas the c.7272G > A variant was absent in both parents, indicating a **de novo** event. Therefore, the patient harbored compound heterozygous *NEB* variants, consistent with an autosomal recessive mode of inheritance (Fig. 2).

In silico analyses using PROVEAN, SIFT, PolyPhen-2 (HVAR and HDIV), MutationTaster, and MaxEntScan predicted that the c.21417 + 3A > G variant disrupts normal RNA splicing and is pathogenic (P), whereas the c.7272G > A variant is a nonsense mutation classified as likely pathogenic. According to the ACMG variant interpretation guidelines, these findings established a molecular diagnosis of *NEB*-*related NM2.*

### 2.11. Final diagnosis

Considering the above findings, we established a final diagnosis of *NEB* gene mutation-related NM2.

### 2.12. Treatment

During the present hospitalization, the patient was diagnosed with NM2. He was prescribed mecobalamin capsules, coenzyme Q10 soft capsules, and idebenone tablets for neurotrophic treatment, in addition to physical rehabilitation therapy to maintain muscle strength and joint mobility. However, symptomatic improvement was limited. The patient was discharged on January 17, 2024, and continued both pharmacological and physical therapy after discharge.

### 2.13. Outcome and follow-up

The patient was scheduled for follow-up every 6 months, and his condition has remained stable.

## 3. Discussion

*NEB*-related NM2 is the most common subtype of NM and accounts for approximately half of all genetically confirmed NM cases.^[6,8]^ As a congenital myopathy, NM exhibits marked genetic and phenotypic heterogeneity, with clinical manifestations ranging from severe neonatal disease with respiratory insufficiency to milder childhood- or adult-onset forms.^[1,2]^ Most pathogenic *NEB* variants are inherited in an autosomal recessive manner and occur as compound heterozygous mutations, including splice-site, frameshift, and nonsense variants.^[3]^ Although *NEB* encodes nebulin, a key structural protein involved in thin filament assembly and sarcomere stability,^[9]^ the relationship between genotype and clinical phenotype remains incompletely understood.^[10]^ Current evidence suggests that disease severity is primarily determined by the combined functional consequences of biallelic pathogenic variants rather than by the parental origin of the mutations. In general, patients harboring 2 truncating or other loss-of-function variants tend to present with more severe phenotypes, whereas combinations involving splice-site or missense variants often exhibit variable clinical manifestations depending on their residual effects on nebulin function. Notably, there is currently no convincing evidence that a de novo *NEB* variant itself is associated with earlier disease onset or greater clinical severity.

In the present case, whole-exome sequencing and parental segregation analysis identified compound heterozygous *NEB* variants consisting of a paternally inherited splice-site variant, c.21417 + 3A > G, and a **de novo** nonsense variant, c.7272G > A, thereby establishing the molecular diagnosis of NM2. The c.21417 + 3A > G variant has been reported predominantly in Chinese and Korean patients and has been proposed as a potential East Asian founder variant.^[2,11]^ This splice-site mutation is predicted to disrupt normal *NEB* mRNA splicing, leading to impaired nebulin function. In contrast, the c.7272G > A variant has not been reported in patients with NM or documented in major population or disease databases, indicating that it is a novel pathogenic variant that further expands the mutational spectrum of *NEB.* Although our patient presented during infancy with a relatively severe phenotype, current evidence suggests that disease severity in *NEB*-related NM is primarily determined by the combined functional effects of biallelic pathogenic variants, residual nebulin activity, and additional genetic or environmental modifiers, rather than by the parental origin of a mutation. Therefore, there is currently no convincing evidence that the **de novo** origin of the c.7272G > A variant itself accounts for the early onset or disease severity observed in this patient. Instead, the **de novo** event should be regarded as the mechanism by which compound heterozygosity was established in this autosomal recessive disorder. This finding highlights the importance of comprehensive molecular testing together with parental segregation analysis, as the absence of 1 pathogenic variant in either parent should not preclude consideration of an autosomal recessive disease. Early molecular diagnosis not only enables accurate disease classification and improves genotype–phenotype characterization but also supports multidisciplinary clinical management, including respiratory surveillance, nutritional support, rehabilitation, orthopedic monitoring, and genetic counseling. Furthermore, although *NEB* variants have also been associated with arthrogryposis multiplex congenita type 6,^[12]^ this diagnosis was excluded in our patient because of the absence of congenital joint contractures or skeletal deformities. Given the limited family data and the lack of functional validation, the pathogenic mechanism and genotype–phenotype correlation of the novel c.7272G > A variant warrant further investigation in additional patients and experimental studies.

The clinical spectrum of NM is broad. Common features include weakness of facial, neck, and proximal limb muscles, along with facial myopathy, high-arched palate, and thoracic abnormalities. As the disease progresses, distal muscles may also be involved, with severe cases leading to respiratory failure.^[6]^ NM2 primarily affects proximal limb muscles, particularly axial and girdle muscles, though distal muscles may also be involved in advanced stages, causing foot drop.^[13]^ In this case, the patient developed symptoms at age 3, with weakness progressing from distal to proximal, accompanied by a long narrow face and high-arched palate, but no ophthalmoplegia, cardiac involvement, or cognitive impairment, which are consistent with classic NM features.^[14,15]^ At age 9, chewing weakness developed, while neck and pharyngeal muscles were relatively preserved, suggesting that NM2 may involve masticatory muscles early in the disease course.

Muscle MRI findings in NM2 vary with disease severity. Mild cases often show abnormalities in the tibialis anterior and soleus, while moderate to severe cases demonstrate widespread involvement of thigh and calf muscles.^[16]^ In this patient, MRI revealed extensive atrophy and fatty infiltration of thigh and calf muscles, particularly the soleus and medial gastrocnemius, which differs from previous reports describing early isolated tibialis anterior involvement.^[11]^ Other studies have reported involvement of the quadriceps and anterior calf muscles.^[17]^ Although no specific genotype–phenotype correlation has been established for MRI patterns, muscle MRI remains valuable in biopsy site selection.^[18]^

The diagnosis of NM relies on muscle biopsy and genetic testing. The characteristic pathological feature is the presence of subsarcolemmal or intrafibrillar rod-shaped structures.^[6]^ Modified Gomori trichrome (MGT) staining is considered the diagnostic gold standard, revealing purple-blue rod-shaped deposits within fibers.^[14,15]^ In this case, only HE staining and electron microscopy were performed, without MGT or Adenosine triphosphatase staining, representing a limitation of the biopsy. HE staining of the gastrocnemius revealed abnormal red deposits and rod aggregation (>2000 nm), consistent with previous reports,^[6,14]^ whereas the triceps brachii showed no abnormalities. Literature indicates that the proportion of rod-positive fibers varies among individuals and muscles, independent of age of onset or disease severity,^[3]^ and that ultrastructural findings may correlate with genotype.^[14]^ Therefore, for suspected NM, when biopsy findings are inconclusive, genetic testing remains the primary diagnostic tool.

At present, there is no effective specific therapy for NM, and management remains supportive and symptomatic. In this patient, symptomatic therapy yielded limited results. Gene therapy is considered the most promising future treatment strategy, though it remains in the exploratory stage. Trials using nebulin-based thin filament disease models have tested supplements such as L-tyrosine, L-carnitine, taurine, and creatine, but none have shown significant improvement in skeletal muscle function. Therefore, treatment for NM remains an urgent challenge.^[19]^

## 4. Conclusions

NM is a rare hereditary myopathy characterized by marked genetic and clinical heterogeneity. In patients presenting with proximal or distal limb weakness, early facial muscle involvement, head-lifting difficulty, and masticatory weakness with preserved swallowing function, NM should be considered in the differential diagnosis. Muscle biopsy, in combination with comprehensive genetic testing, remains essential for establishing an accurate diagnosis.

This case expands the mutational spectrum of *NEB*-related NM by identifying a rare combination of an inherited splice-site variant and a **de novo** nonsense variant, resulting in compound heterozygosity. Although *NEB*-related NM is inherited in an autosomal recessive manner, this case highlights that a **de novo** pathogenic variant may represent 1 of the 2 disease-causing alleles and therefore should be considered during molecular diagnosis and parental segregation analysis. Comprehensive genetic testing not only enables a definitive molecular diagnosis but also improves genotype–phenotype characterization, facilitates multidisciplinary clinical management, and provides a robust basis for genetic counseling and recurrence-risk assessment.

Several limitations should be acknowledged. MGT staining was not performed on the muscle biopsy specimen, and detailed clinical evaluation of additional family members was unavailable. Further studies involving larger patient cohorts and functional analyses of novel NEB variants are warranted to better elucidate genotype–phenotype correlations and their implications for disease pathogenesis and precision medicine in NM.

## Author contributions

**Conceptualization:** Yan-Fang Zhang, Guo-De Wu, Jian-Fei Wang

**Data curation:** Yan-Fang Zhang, Guo-De Wu, Ya-Zhen Hao, Wen-Juan Li, Xiao-Ling Qian.

**Formal analysis:** Yan-Fang Zhang, Ya-Zhen Hao, Wen-Juan Li, Xiao-Ling Qian, Jian-Fei Wang.

**Investigation:** Yan-Fang Zhang, Guo-De Wu, Ya-Zhen Hao, Wen-Juan Li, Xiao-Ling Qian.

**Methodology:** Yan-Fang Zhang, Guo-De Wu, Ya-Zhen Hao, Wen-Juan Li, Xiao-Ling Qian.

**Writing – original draft:** Yan-Fang Zhang, Guo-De Wu, Ya-Zhen Hao, Wen-Juan Li, Xiao-Ling Qian.

**Writing – review & editing:** Yan-Fang Zhang, Guo-De Wu, Ya-Zhen Hao, Wen-Juan Li, Xiao-Ling Qian, Jian-Fei Wang.

## References

[1] Wallgren-Pettersson C Laing NG. Report of the 70th ENMC International Workshop: nemaline myopathy, 11-13 June 1999, Naarden, The Netherlands. Neuromuscul Disord. 2000;10:299–306. 10.1016/s0960-8966(99)00129-7 10838258

[2] Wang Q Hu Z Chang X. Mutational and clinical spectrum in a cohort of Chinese patients with hereditary nemaline myopathy. Clin Genet. 2020;97:878–89. 10.1111/cge.13745 32222963

[3] Laitila J Wallgren-Pettersson C. Recent advances in nemaline myopathy. Neuromuscul Disord. 2021;31:955–67. 10.1016/j.nmd.2021.07.012 34561123

[4] Ogasawara M Nishino I. A review of core myopathy: central core disease, multiminicore disease, dusty core disease, and core-rod myopathy. Neuromuscul Disord. 2021;31:968–77. 10.1016/j.nmd.2021.08.015 34627702

[5] Kiiski KJ Lehtokari VL Vihola AK. Dominantly inherited distal nemaline/cap myopathy caused by a large deletion in the nebulin gene. Neuromuscul Disord. 2019;29:97–107. 10.1016/j.nmd.2018.12.007 30679003

[6] Haidong L Yin L Ping C. Clinico-pathological and gene features of 15 nemaline myopathy patients from a single Chinese neuromuscular center. Acta Neurol Belg. 2024;124:91–9. 10.1007/s13760-023-02333-8 PMC10874337 37525074

[7] Richards S Aziz N Bale S; ACMG Laboratory Quality Assurance Committee. Standards and guidelines for the interpretation of sequence variants: a joint consensus recommendation of the American College of Medical Genetics and Genomics and the Association for Molecular Pathology. Genet Med. 2015;17:405–24.

[8] Claeys KG. Congenital myopathies: an update. Dev Med Child Neurol. 2020;62:297–302. 10.1111/dmcn.14365 31578728

[9] Wen Q Chang X Guo J. A childhood-onset nemaline myopathy caused by novel heterozygote variants in the nebulin gene with literature review. Acta Neurol Belg. 2020;120:1351–60. 10.1007/s13760-019-01230-3 31696431

[10] Sewry CA Laitila JM Wallgren-Pettersson C. Nemaline myopathies: a current view. J Muscle Res Cell Motil. 2019;40:111–26. 10.1007/s10974-019-09519-9 PMC6726674 31228046

[11] Wunderlich G Brunn A Daimagüler HS. Long term history of a congenital core-rod myopathy with compound heterozygous mutations in the Nebulin gene. Acta Myol. 2018;37:121–7. PMC6060425 30057997

[12] Feingold-Zadok M Chitayat D Chong K. Mutations in the NEB gene cause fetal akinesia/arthrogryposis multiplex congenita. Prenat Diagn. 2017;37:144–50. 10.1002/pd.4977 27933661

[13] Wallgren-Pettersson C Sewry CA Nowak KJ Laing NG. Nemaline myopathies. Semin Pediatr Neurol. 2011;18:230–8. 10.1016/j.spen.2011.10.004 22172418

[14] Ryan MM Ilkovski B Strickland CD. Clinical course correlates poorly with muscle pathology in nemaline myopathy. Neurology. 2003;60:665–73. 10.1212/01.wnl.0000046585.81304.bc 12601110

[15] Laflamme N Lace B Thonta Setty S. A homozygous deep intronic mutation alters the splicing of nebulin gene in a patient with nemaline myopathy. Front Neurol. 2021;12:660113. 10.3389/fneur.2021.660113 PMC8239344 34211429

[16] Romero NB Sandaradura SA Clarke NF. Recent advances in nemaline myopathy. Curr Opin Neurol. 2013;26:519–26. 10.1097/WCO.0b013e328364d681 23995272

[17] Chen P Qu QQ Cui WH. Clinical characteristics of childhood-onset nemaline myopathy caused by novel compound heterozygous mutations in the NEB gene (a case report). J Clin Neurol. 2021;2:1004.

[18] Ennis J Dyment DA Michaud J McMillan HJ. Congenital nemaline myopathy: the value of magnetic resonance imaging of muscle. Can J Neurol Sci. 2015;42:338–40. 10.1017/cjn.2015.59 26348902

[19] Sztal TE McKaige EA Williams C Oorschot V Ramm G Bryson-Richardson RJ. Testing of therapies in a novel nebulin nemaline myopathy model demonstrate a lack of efficacy. Acta Neuropathol Commun. 2018;6:40. 10.1186/s40478-018-0546-9 PMC5977763 29848386

